# Tensin1 expression and function in chronic obstructive pulmonary disease

**DOI:** 10.1038/s41598-019-55405-2

**Published:** 2019-12-12

**Authors:** Panayiota Stylianou, Katherine Clark, Bibek Gooptu, Dawn Smallwood, Christopher E. Brightling, Yassine Amrani, Katy M. Roach, Peter Bradding

**Affiliations:** 1 Department of Respiratory Sciences, University of Leicester, UK, Institute of Lung Health and NIHR Leicester BRC-Respiratory, Leicester, UK; 2 Department of Molecular and Cell Biology, University of Leicester, Leicester, UK; 3 Leicester Institute of Structural and Chemical Biology, University of Leicester, Leicester, UK; 4 Faculty of Health and Life Sciences, De Montfort University, Leicester, UK

**Keywords:** Mechanisms of disease, Chronic obstructive pulmonary disease

## Abstract

Chronic obstructive pulmonary disease (COPD) constitutes a major cause of morbidity and mortality. Genome wide association studies have shown significant associations between airflow obstruction or COPD with a non-synonymous SNP in the TNS1 gene, which encodes tensin1. However, the expression, cellular distribution and function of tensin1 in human airway tissue and cells are unknown. We therefore examined these characteristics in tissue and cells from controls and people with COPD or asthma. Airway tissue was immunostained for tensin1. Tensin1 expression in cultured human airway smooth muscle cells (HASMCs) was evaluated using qRT-PCR, western blotting and immunofluorescent staining. siRNAs were used to downregulate tensin1 expression. Tensin1 expression was increased in the airway smooth muscle and lamina propria in COPD tissue, but not asthma, when compared to controls. Tensin1 was expressed in HASMCs and upregulated by TGFβ1. TGFβ1 and fibronectin increased the localisation of tensin1 to fibrillar adhesions. Tensin1 and α-smooth muscle actin (αSMA) were strongly co-localised, and tensin1 depletion in HASMCs attenuated both αSMA expression and contraction of collagen gels. In summary, tensin1 expression is increased in COPD airways, and may promote airway obstruction by enhancing the expression of contractile proteins and their localisation to stress fibres in HASMCs.

## Introduction

Chronic obstructive pulmonary disease (COPD) constitutes a major cause of morbidity and mortality with more than 200 million people affected worldwide^1,2^. It is characterised by irreversible airway narrowing which limits airflow, resulting in breathlessness and in severe cases, respiratory failure^3^. The most common cause is tobacco smoking^4^. COPD is characterised by airway remodeling that involves thickening of the airway wall and the airway smooth muscle (ASM) layer, hypersecretion of mucous and metaplasia of epithelial cells^5,6^. Airway wall thickness results from an increase in each of the airway wall compartments, including the epithelium, ASM and lamina propria^7^. Under normal conditions ASM has a critical role in regulating the tone of the airways and distribution of airflow by maintaining a balance between contractile and dilatory processes^8^. In COPD, changes occur in airway smooth muscle, notably in the small airways^7,9^, and ASM cells mediate deposition of collagens, fibronectin and laminin in extracellular matrix^10,11^.

Recent evidence suggests that gene-environment interactions constitute a major factor for the development of COPD^12^. Genome wide association studies (GWAS) have shown a significant association between a non synonymous single nucleotide polymorphism (SNP) in the coding region of the tensin1 gene (*TNS1*) and COPD^13^, as well as airflow obstruction^14^ and early childhood wheeze^15^. Tensin1 is a 220 kDa cytoplasmic phosphoprotein, which localises to integrin-mediated focal and fibrillar adhesions^16^. These adhesions provide a bi-directional link between the extracellular matrix and the cytoskeleton^17^. A phosphotyrosine domain in the C-terminal region of tensin1 facilitates an interaction with the α5β1 integrin, which itself binds to the extracellular matrix protein fibronectin. Furthermore, an actin-binding domain is located on the N-terminal of tensin1 enabling interaction with the actin filaments, and in particular the F-actin protein^18,19^. The interactions of tensin1 with these intracellular and extracellular molecules contribute to its involvement in cell signaling and function. However, the pattern of expression and (patho)physiological role of tensin1 in human airways is not known.

We have therefore characterised the localisation of tensin1 and its cellular function in the context of COPD. We have further investigated whether the TNS1 SNP (2q35, rs257114 C > T, resulting in R1197W amino acid substitution) could play a pathogenic role in COPD.

## Results

### Clinical characteristics

The clinical characteristics of the controls, COPD and asthma subjects used for immunohistochemistry and cell culture studies are summarised in Tables 1–3.

**Table 1:** Clinical characteristics of non-COPD controls and COPD subjects used for immunohistochemistry.

	Lung resections		Statistical analysis
	non-COPD Lung resections (n = 11)	COPD Lung resections (n = 13)	
Age (years)	66.9 ± 8.6	74.3 ± 9.4	p = 0.0284
Gender (M/F)	8/3	9/4	NS
FEV_1_(% predicted)	83.3 ± 9.5	68.2 ± 10.9	p = 0.0004
FEV_1_/FVC ratio (%)	79.8 ± 6.7	60.0 ± 8.8	p < 0.0001
Smoking (pack years)	15.5 ± 15.3	34.7 ± 16.6	p = 0.0024
Inhaled corticosteroid dose (µg)	0	0	NS
Severity	NA	GOLD 1 (2) & GOLD 2 (11)	NA

Data are mean ± SD unless otherwise stated (SD: Standard deviation, FEV1: forced expiratory volume at second 1, FVC: forced vital capacity, CI: confidence interval, NA: not applicable; NS: non-significant).

**Table 2:** Clinical characteristics of healthy controls and asthma subjects used for immunohistochemistry.

	Bronchial biopsies		Statistical analysis
	Healthy Biopsies (n = 9)	Asthmatic Biopsies (n = 10)	
Age (years)	31.6 ± 15.9	37.7 ± 12.4	NS
Gender (M/F)	5/4	4/6	NS
FEV_1_(% predicted)	102.2 ± 11.6	79.4 ± 13.2	p = 0.0117
FEV_1_/FVC ratio (%)	81.4 ± 7.4	66.9 ± 8.4	p = 0.0180
Smoking (pack years)	0.4 ± 1.2	0.9 ± 2.3	NS
PC20 methacholine (mg/ml), geometric mean (95% CI)	>16	6.8 (2.9–10.5)	p = 0.0006
Reversibility to β-agonist (%)	1.6 ± 4.9	19.7 ± 25.1	p = 0.0465
Serum IgE (KU/L), geometric mean (95% CI)	43.8 (16.2–71.4)	172.1 (97.5–246.6)	p = 0.0054
Inhaled corticosteroid dose (µg)	0	192.9 ± 148.4	p = 0.0008
Severity	NA	Mild (3), Moderate (2) & Severe (5)	NA
Age of onset	NA	20.3 ± 14.3	NA

Data are mean ± SD unless otherwise stated (SD: Standard deviation, FEV_1_: forced expiratory volume at second 1, FVC: forced vital capacity, CI: confidence interval, NA: not applicable; NS: non-significant).

**Table 3:** Clinical characteristics of healthy controls, COPD and asthmatic subjects used for cell culture studies.

	Human airway smooth muscle cells			Statistical analysis
	Healthy	COPD	Asthma	
Age (years)	42.5 ± 4.7	69.7 ± 1.5	44.5 ± 5.2	p = 0.0001
Gender (M/F)	5/7	7/3	6/3	NS
FEV_1_(% predicted)	95.6 ± 3.2	54.4 ± 5.4	96.5 ± 4.8	p < 0.0001
FEV_1_/FVC ratio (%)	80.2 ± 1.5	53.7 ± 3.1	79.1 ± 2.4	p < 0.0001
Pack Years (y)	1.7 ± 0.9	41.7 ± 4.5	6.1 ± 5.4	p < 0.0001
PC20 methacholine (mg/ml), geometric mean (95% CI)	9.3 (3.9–14.6)	ND	4.3 (1.3–7.3)	p = 0.0463
Reversibility to β-agonist (%)	NA	ND	−1.3 ± 2.4	NA
Serum IgE (KU/L), geometric mean (95% CI)	4.9 (4.2–5.5)	ND	498 (396.3–599.6)	p < 0.0001
Inhaled corticosteroid dose (µg)	0	500	633.3 ± 190.5	p = 0.0012
Severity	NA	GOLD 1 – GOLD 4	GINA 1 – GINA 5	NA
Age of Onset	NA	57 ± 2.3	31.5 ± 3.6	p = 0.0005

Data are mean ± SD unless otherwise stated (SD: Standard Deviation, FEV1: forced expiratory volume at second 1, FVC: forced vital capacity, CI: confidence interval, NA: not applicable, ND: not done & NS: non-significant).

### Tensin1 immunoreactivity is increased in both the airway smooth muscle and lamina propria in patients with COPD but not asthma

**Figure 1:**
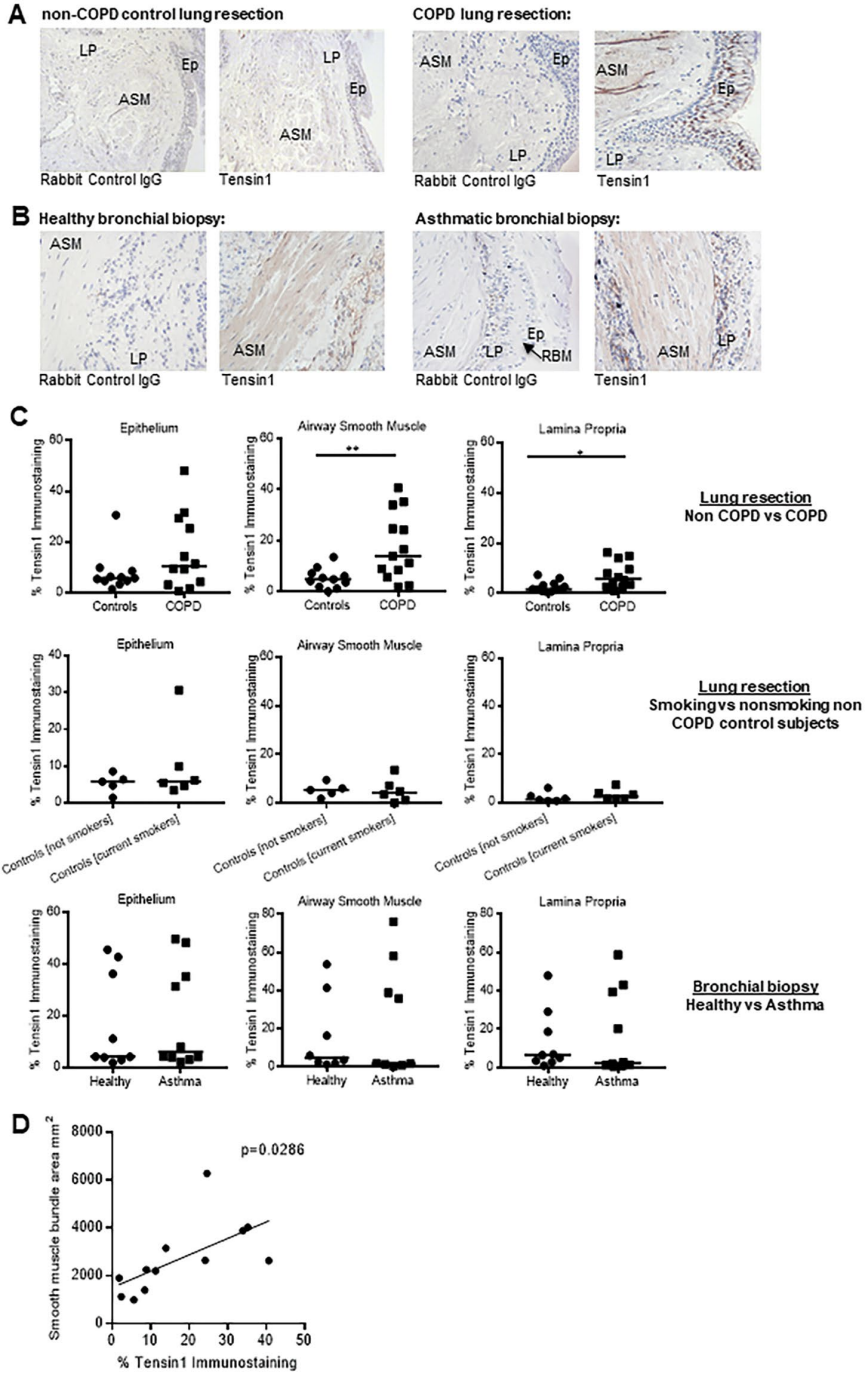
Tensin1 immunostaining in non-disease controls and COPD lung resection and healthy and asthmatic bronchial biopsies. (**A**) Examples of tensin1 and isotype control immunostaining in COPD (n = 13) and non-COPD lung resections (n = 11) (EP: epithelium; ASM: airway smooth muscle; LP: lamina propria). **(B)** Examples of tensin1 and isotype control immunostaining in bronchial biopsies from patients with asthma (n = 10) and healthy controls (n = 9) (EP: epithelium; ASM: airway smooth muscle; LP: lamina propria, RBM: reticular basement membrane). (**C**) The extent of tensin1 immunostaining in airway epithelium, airway smooth muscle and lamina propria analysed using threshold measurements. Increased tensin1 immunostaining was observed in the airway smooth muscle (**p = 0.007) and lamina propria (*p = 0.012) in COPD subjects when compared to non-COPD controls (Mann-Whitney unpaired non parametric test). Tensin1 immunostaining in lung resections was not affected by the smoking status of non-COPD control individuals. **(D)** Tensin1 immunostaining in COPD subjects was positively correlated with the smooth muscle bundle area, calculated by Pearson’s correlation (R = 0.6286, p = 0.0286).

To identify the expression and cellular localisation of tensin1 in human airways *in vivo*, tissue was immunostained for tensin1. Immunostaining was performed on non-COPD control (n = 11) and COPD (n = 13) airway tissue from lung resections, and healthy (n = 9) and asthmatic (n = 10) bronchial biopsies. Positive tensin1 staining was observed in the apical epithelium, ASM bundles and lamina propria (Fig. 1A,B). Quantification of tensin1 immunostaining was performed using a thresholding technique as previously described^20^. A significant increase in tensin1 immunostaining was detected in both the lamina propria and ASM in COPD compared to non-COPD control tissue (Fig. 1C). There was no significant difference between COPD and control airway epithelium (Fig. 1C).

Since smoking is the most common causative factor for COPD and the COPD group had a greater smoking history than non-COPD controls, we examined the correlation of tensin1 and tobacco smoking. The extent of tensin1 immunostaining was not affected by the smoking status of non-COPD control individuals (Fig. 1C), and there was no correlation between tensin1 immunostaining and smoking in the COPD subjects in any compartment (p > 0.05 for all comparisons). The COPD patients were also a little older than the control group (Table 1) but tensin1 immunostaining was not correlated with age in any tissue compartment in either non-COPD controls or COPD subjects (p > 0.05 for all comparisons).

Tensin1 immunostaining was not increased in any airway compartment in asthma (Fig. 1C). Tensin1 immunostaining correlated positively with the area of the smooth muscle bundles in COPD subjects (R = 0.6286, p = 0.0286) (Fig. 1D). Isotype control staining was negative. Specificity of the antibody for tensin1 was confirmed by immunoprecipitation (Figure S1A in the Supplementary Information).

### Human airway smooth muscle cells express tensin1 mRNA, which is up-regulated by TGFβ1

As tensin1 was most strongly increased in the ASM in COPD subjects when compared to controls, its role in cultured HASMCs was studied further. We studied expression at the mRNA and protein level in HASMCs derived from healthy, asthmatic and COPD airway tissue collected at bronchoscopy, basally and following stimulation with TGFβ1 or fibronectin.

**Figure 2:**
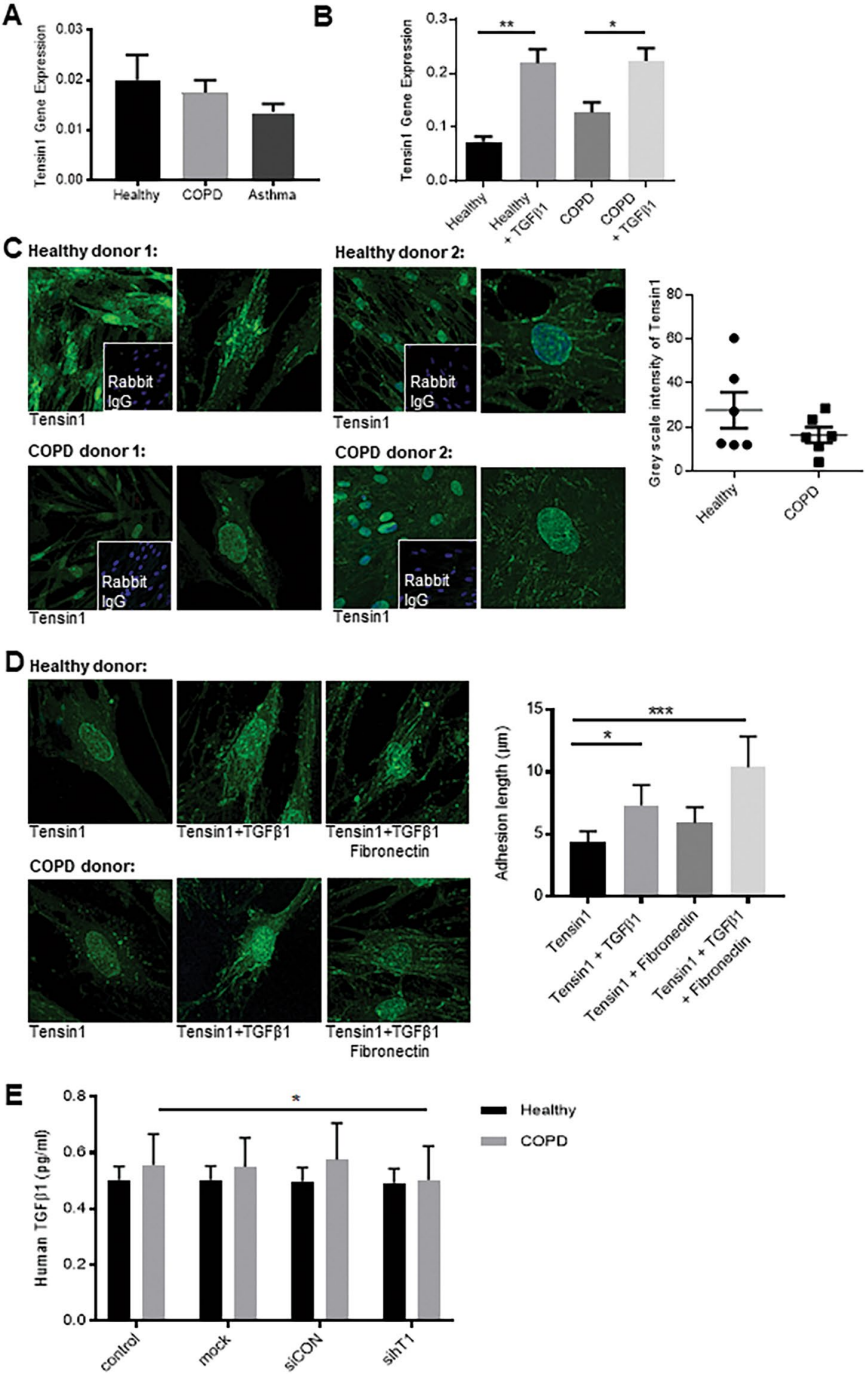
Tensin1 mRNA and protein expression in HASMCs in health and disease and the effect of TGFβ1 and fibronectin. (**A**) Quantitative Reverse Transcription -PCR (qRT-PCR) was used to quantify tensin1 mRNA expression in HASMCs isolated from healthy donors and patients with COPD or asthma using the 2^-(ΔCt) method (n = 7 of each, Mean ± SEM). **(B)** Tensin1 mRNA expression in human airway smooth muscle cells (HASMCs) was increased following TGFβ1-dependent stimulation in both COPD (n = 3) (*p = 0.0418) and healthy (n = 3) (**p = 0.0042) donors (Tukey’s multiple comparison test as part of one-way ANOVA, one-way ANOVA test: **p = 0.0019). **(C)** HASMC tensin1 immunofluorescent staining was measured by grey scale intensity in n = 6 healthy and n = 6 COPD subjects. No difference was observed in the grey scale intensity of tensin1 immunostaining in the two phenotypes (Mean ± SEM). **(D)** Cells were stimulated with TGFβ1 and/or fibronectin. Stimulation with TGFβ1 and fibronectin did not significantly increase the grey scale intensity of tensin1. However, the length of fibrillar adhesions was significantly increased in HASMCs stimulated with TGFβ1 alone (*p = 0.0386) and TGFβ1 + fibronectin (***p = 0.0002) (Dunnett’s multiple comparison test as part of one-way ANOVA, one-way ANOVA test: ***p = 0.0004). Data shown is pooled COPD and healthy donors which did not differ (n = 3 of each, Mean ± SEM). **(E)** Cells were transfected with siRNA directed against tensin1 and supernatants were assessed for TGFβ1 expression using ELISA in both COPD (n = 4) and healthy donors (n = 4) (Mean ± SEM). Tensin1 depleted HASMC supernatants derived from COPD subjects had a small but significant reduction of TGFβ1 secretion when compared to controls (*p = 0.0477) (paired t-test).

Tensin1 mRNA was expressed at high levels in HASMCs, with no differences detected between patients with COPD or asthma and healthy controls (Fig. 2A). Tensin1 mRNA expression by HASMCs was significantly upregulated by TGFβ1 stimulation and to a similar extent in both healthy (n = 3) and COPD (n = 3) subjects (Fig. 2B). Overall, *in vitro*, tensin1 protein expression was similar between HASMCs derived from healthy and COPD tissue (Fig. 2C) under basal conditions. As tensin1 facilitates integrin binding to fibronectin, we examined whether culturing HASMCs on fibronectin altered tensin1 distribution, but there was no significant change (Fig. 2D). However, stimulating HASMCs cultured on plastic or fibronectin with TGFβ1 significantly increased the length of fibrillar adhesions defined by tensin1 immunostaining in HASMCs derived from both healthy and COPD donors (Fig. 2D). In contrast to the transcriptomic data, and findings in tissue *ex vivo*, these stimuli did not significantly increase overall tensin1 protein levels in the HASMCs, assessed by immunostaining and greyscale analysis (Supplementary Fig. S1B).

siRNA transfection achieved around 92% silencing of tensin1 mRNA after 48 hours, as quantified by the 2^−(ΔCt)^ method (n- = 3) (Supplementary Fig. S1C). Silencing of tensin1 did not have any effect on the survival or proliferation of HASMCs in culture (n = 4 healthy and n = 4 COPD) assessed by the MTS assay (Supplementary Fig. S2A). Tensin1 silencing was associated with a small but significant reduction in TGFβ1 secretion in COPD-derived HASMCs, but this was not evident in supernatants when HASMCs derived from healthy subjects were used (Fig. 2E).

Taken together, these data indicate that the apparent increase in cellular tensin1 protein signal in COPD ASM *in vivo* may be driven by interactions with the abnormal matrix in disease rather than inherent changes within the cells in the disease state. TGFβ1 and fibronectin stimulation does not appear to account for this, but they do alter the cellular distribution of the protein to associate more with fibrillar adhesions of greater length in HASMCs from both healthy and COPD tissue.

### Tensin1 stimulates αSMA expression and interacts with it in HASMCs, and mediates contraction

**Figure 3:**
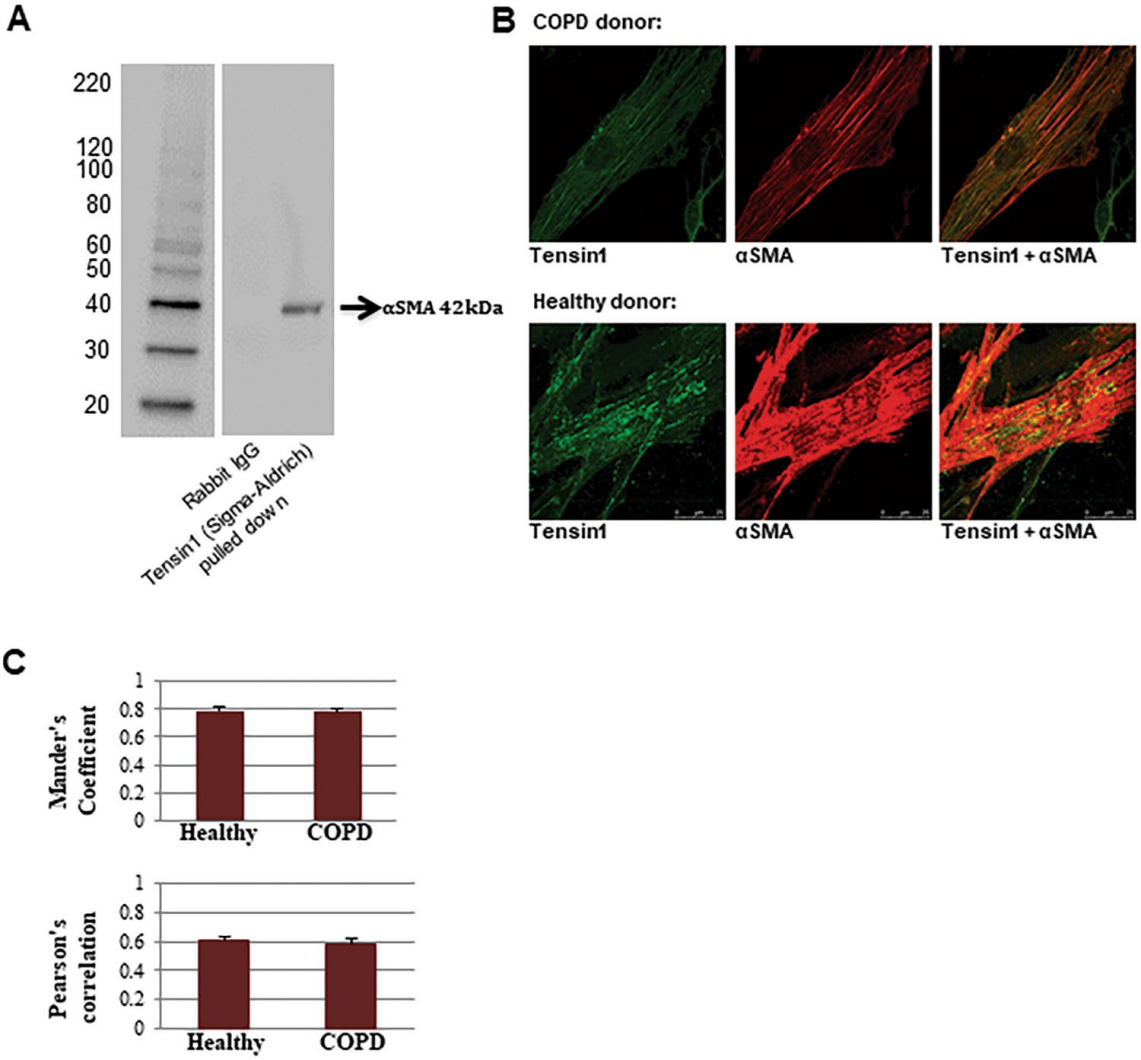
Tensin1 co-localises and correlates with αSMA in HASMCs. (**A)** Co-immunoprecipitation was carried out to investigate the interaction of tensin1 and αSMA. Tensin1 immunoprecipitates were analysed by western blotting analysis using an αSMA antibody. A band of 42 kDa was detected suggesting a physical interaction between tensin1 and αSMA. **(B)** Confocal immunofluorescent staining demonstrating co-localisation of tensin1 (green) and αSMA (red) in HAMSCs. **(C)** Mander’s overlap coefficient and Pearson’s correlation were calculated to confirm association of the two proteins on n = 3 healthy and n = 3 COPD subjects (Mean ± SEM).

We next assessed whether the increased length of fibrillar adhesions following TGFβ1 stimulation related to increased interactions with, and/or increased expression of, αSMA in HASMCs derived from healthy controls (n = 3) and COPD individuals (n = 3). Colocalisation and close physical association of tensin1 and αSMA was demonstrated by co-immunoprecipitation (co-IP, Fig. 3A) and confocal microscopy (Fig. 3B**)**. In all conditions, strong co-localisation of tensin1 and αSMA immunofluorescence was observed (Fig. 3B). Quantification of the degree of colocalisation by overlap analysis (Mander’s overlap coefficient = 0.8 and Pearson’s correlation~0.6) revealed strong spatial colocalisation between the two proteins (Fig. 3C).

**Figure 4:**
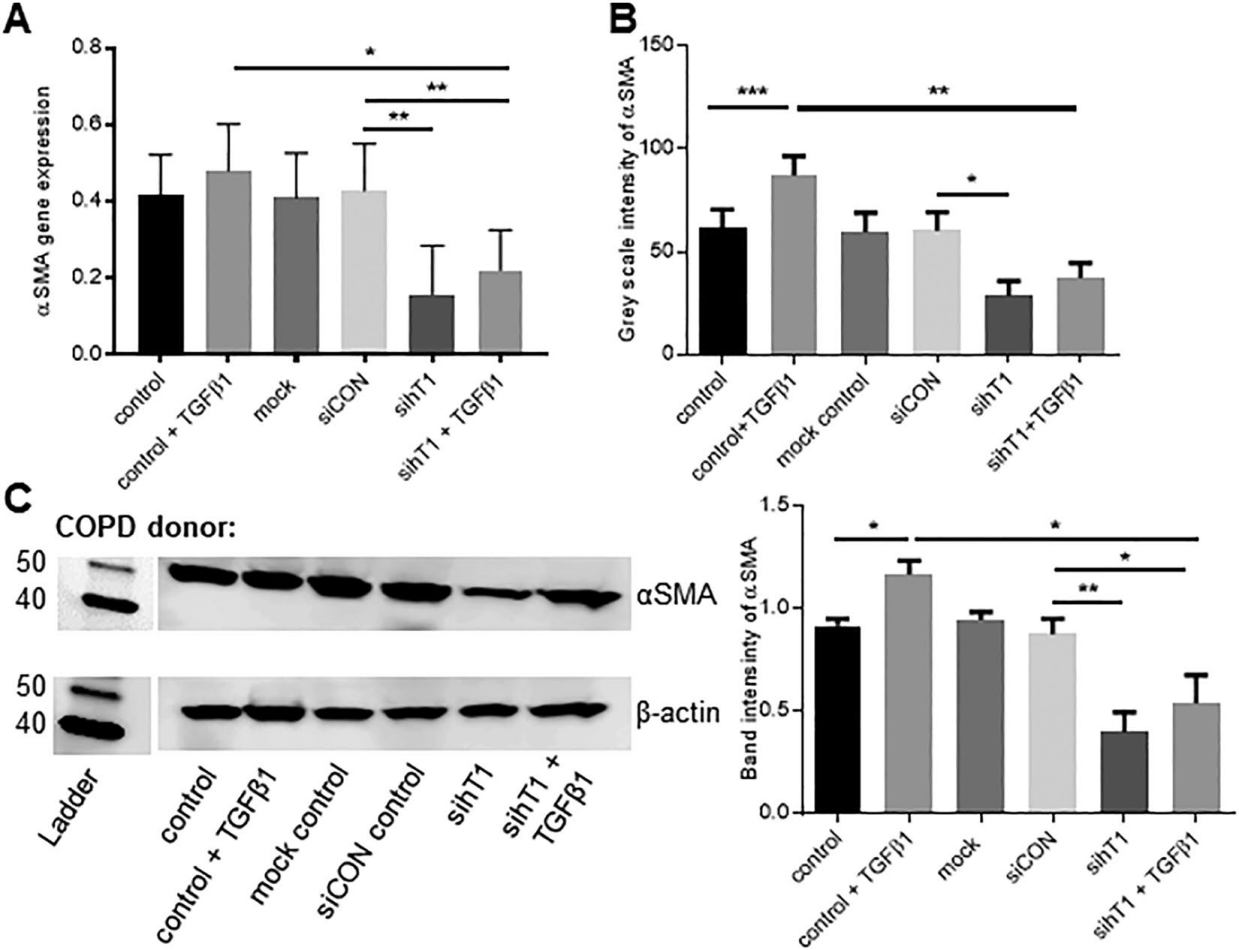
Reduced αSMA mRNA and protein expression in tensin1-depleted HASMCs. Tensin1 siRNA-transfected and control (untreated cells, transfection reagent alone, non-targeting siRNA control) HASMCs derived from healthy individuals and COPD patients were analysed for αSMA mRNA and protein expression. Cells were also stimulated with TGFβ1 to examine its role in αSMA expression after silencing tensin1. (**A**) The effects of depleting tensin1 on HASMC αSMA mRNA expression was examined using qRT-PCR on n = 4 COPD donors. αSMA mRNA expression was quantified using the 2^−(ΔCt)^ method (Mean ± SEM). Silencing of tensin1 resulted in significant downregulation of αSMA mRNA in HASMCs when compared to control (p = 0.0011 by repeated measures ANOVA). *p < 0.05, **p < 0.01 by Sidaks multiple comparison test (Mean ± SEM). **(B)** Cells were transfected with siRNA directed against tensin1 and assessed for αSMA expression using immunofluorescence analysis. Tensin1-depleted HASMCs had a significantly lower intensity of αSMA when compared to controls both in the absence or presence of TGFβ1 (p = 0.0002 by repeated measures ANOVA). **p < 0.05, ** p < 0.01, *** p < 0.001 by Sidaks multiple comparison test. Mean ± SEM. Data in B are pooled COPD (n = 3) and healthy donors (n = 3) which did not differ. **(C)** The effects of depleting tensin1 on HASMC αSMA protein expression was also confirmed using western blot analysis (p = 0.0006 by repeated measures ANOVA). *p < 0.05, **p < 0.01 by Sidaks multiple comparison test. Mean ± SEM. The left panel shows a representative western blot. Data in the right panel are pooled COPD (n = 4) and healthy donors (n = 4) which did not differ.

Tensin1 silencing significantly reduced αSMA expression at the mRNA (Fig. 4A) and protein levels (Figs. 4B,C, S2B) in both non-stimulated and TGFβ1-stimulated HASMCs derived from healthy and COPD lung tissue. There were no apparent differences relating to the health/disease status of the original lung tissue.

**Figure 5:**
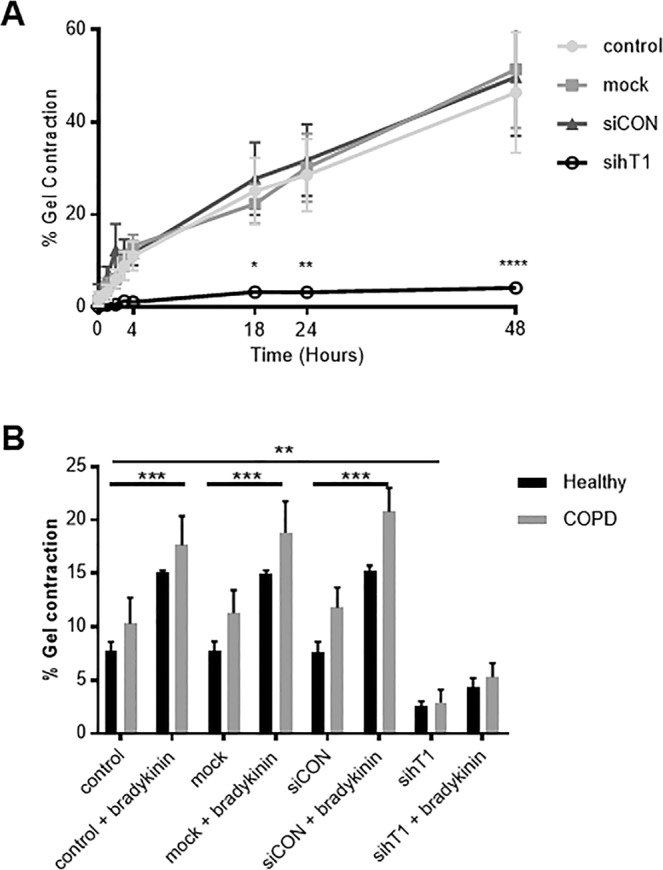
Collagen gel contraction by HASMC is dependent on tensin1. (**A)** Cells were transfected with siRNA directed against tensin1 and incubated within 3D collagen gels. The extent of spontaneous collagen gel contraction was recorded at 4, 18, 24 and 48 hours. Quantification of collagen gel contraction using gel area measurement was performed (n = 8, Mean ± SEM). Data shown are pooled COPD (n = 4) and healthy donors (n = 4) which did not differ. HASMCs transfected with tensin1 siRNA SMARTpool showed a greatly reduced ability to contract, with significant differences compared to controls at 18 (*p = 0.0118), 24 (**p = 0.0027) and 48 hours (****p = 0.0001) (Dunnett’s multiple comparison test as part of two-way ANOVA). **(B)** Cells were stimulated with bradykinin and collagen gel contraction was assessed on COPD (n = 4) and healthy donors (n = 4) (Mean ± SEM) after 18 hours. Contraction in tensin1 depleted HASMCs was greatly reduced (**p = 0.0033). The extent of collagen gel contraction following bradykinin stimulation was significantly increased in non- (***p = 0.0009), mock (***p = 0.0009) and siCON (***p = 0.0001) transfected cells, when compared to spontaneous contraction. Tensin1-depleted HASMCs did not respond significantly to bradykinin, when compared to controls (Tukey’s multiple comparison test as part of two-way ANOVA).

To assess the mechanistic significance of tensin1- αSMA interactions, collagen gel contraction assays^21,22^ were performed to study HASMC contraction. A representative example of these experiments can be seen in Fig. S3 (Supplementary Information). These studies indicated that tensin1 depletion greatly reduced HASMC contraction, with no difference between HASMCs derived from healthy (n = 4) and COPD (n = 4) subjects (Fig. 5A). Collagen gel contraction increased significantly following stimulation with the contractile agonist bradykinin in controls (untreated cells, transfection reagent alone, siCON control), but not in cells subjected to tensin1 knockdown (Figs. 5B and S4 [Supplementary Figure]). Taken together these data indicate that tensin1 interactions with αSMA increase αSMA expression, are increased in association with increased fibrillar adhesion length in HASMCs following TGFβ1 stimulation, and that these responses enhance contraction, irrespective of disease status.

### Expression of the R1197 and R1197W tensin1 variants in healthy, asthmatic and COPD HASMCs

**Figure 6:**
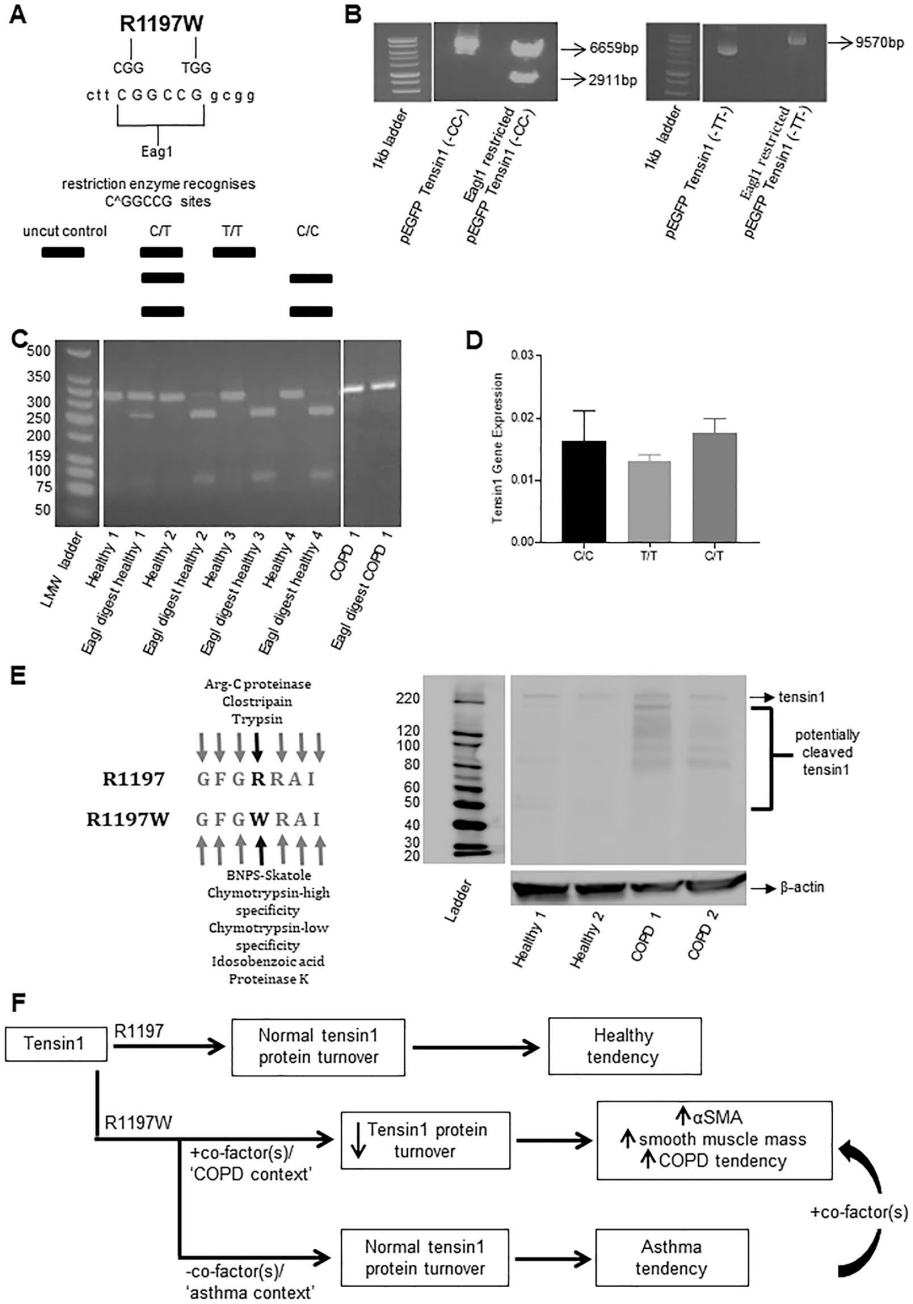
The distribution of C and T alleles generating amino acid 1997 of tensin1 and schematic illustrating potential role of tensin1 in disease. (**A)** A diagram illustrating the base substitution at the site of the variant nucleotide base, the Eag1 restriction site formation in the presence of C versus T, and the different tensin1 genotypes evident on digested PCR products. **(B)** pEGFP-C1 constructs with the tensin1 variants were used as controls to confirm validity of the RFLP technique. **(C)** An agarose gel image illustrating the different tensin1 phenotypes in HAMSCs. Lane 1 = ladder. Lane 3 = Heterozygous, Lane 5, 7 and 9 = homozygous (-CC-) (encodes R1197 tensin1) and Lane 11 = homozygous (-TT-) (encodes R1197W tensin1). **(D)** Tensin1 mRNA expression was assessed between the different genotypes that segregated with health and disease. **(E)** A western blotting analysis illustrating differential cleavage of tensin1 in COPD subjects when compared to healthy controls (left). ‘PeptideCutter’ prediction tool showing differential cleavage at the site of mutation (right). **(F)** A schematic illustrating our hypothesis that the R1197W mutation and COPD-specific co-factors together modulate tensin1 degradation and thereby affect αSMA expression, and ASM mass.

Having characterised tensin1 expression in COPD airway tissue and TGFβ1-stimulated HASMCs, and confirmed its interaction with αSMA was a key mediator of HASMC contraction, we then assessed the pathogenic potential of the R1197W mutation. RFLP analysis was used to genotype subjects for the presence of the polymorphism as its presence results in disruption of the formation of a restriction enzyme site (Fig. 6A). pEGFP constructs containing the full length of *TNS1* cDNA with the C or T alleles were used as controls to validate the method (Fig. 6B). In Fig. 6C, the PCR-RFLP detection assay can be seen demonstrating the presence of the C or T alleles in HASMCs from healthy or COPD subjects. RFLP analysis revealed the predominant presence of the C/C homozygous genotype (which generates R1197) in healthy controls (11 of the 14 tested), and the heterozygote C/T or homozygous T/T (R1197W) genotype in COPD donors (12 of the 12 tested) (p < 0.0001) (Table 4). This indicates a strong link between the R1197W amino acid change and COPD, consistent with the GWAS signal (p = 1.11 × 10–12) [14]. In patients with asthma ranging from mild to severe, 8 of 9 samples tested were the heterozygote C/T or homozygous T/T genotype, similar to COPD **(**Table 4**)**. Taken together, these data suggest the mutation is a risk factor for obstructive airway disease as a whole, but the lack of increased tensin1 protein expression in asthmatic airways suggests different handling of tensin1 in these two conditions despite a frequently shared genotype.

**Table 4:** Genotype profile of healthy and diseased subjects for tensin1.

Group	Number	Genotype frequency, no. (%)	
		CC	CT or TT
Healthy	14	11 (75%)	2 + 1 (25%)
COPD	12	—	11 + 1 (100%)
Asthma	9	1 (11.11%)	6 + 2 (88.89%)

HASMCs from 14 healthy controls, 12 COPD subjects and 9 asthmatic subjects were genotyped for the two tensin1 variants. RFLP revealed the presence of the homozygous C/C (R1197) variant only in healthy controls, while COPD donors were predominantly T/C heterozygotes or occasionally T/T homozygotes (p < 0.0001). The distribution in asthma was similar to COPD (p = 0.0075).

qRT-PCR demonstrated similar tensin1 mRNA expression levels between the different *TNS1* genotypes in HASMCs (Fig. 6D).

HASMCs were transfected with pEGFP constructs containing full length *TNS1* cDNA with either the C or T allele and both constructs revealed similar transfection efficiency. Overexpression of tensin1 using pEGFP constructs containing the full length of *TNS1* cDNA with the C or T alleles also did not have any effect on survival or proliferation (Supplementary Fig. S5B). Since the *TNS1* SNP does not appear to affect transcription or translation of the TNS1 gene product tensin1, we further hypothesised that the mutation’s effects were likely to be mediated by differential turnover. Tensins are known to be highly susceptible to proteolysis^23^. Consistent with this, the mutation changes the predicted proteolytic sensitivities of tensin1 as assessed by ‘PeptideCutter’ prediction tool (Fig. 6E). Moreover, western blot analysis of HASMC lysates derived from healthy and COPD patient tissue provides some support for this hypothesis with a differential cleavage profile observed between the two (Fig. 6E). Much of the tensin1 protein from COPD-derived HASMCs appeared to be proteolytically modified, whilst that from HASMCs derived from healthy tissue appeared to be present solely in its full-length, 220 kDa form (Fig. 6E).

## Discussion

Modern genetic approaches such as GWAS can provide powerful insight in understanding disease by identifying novel pathogenic mutations and highlighting important pathways that may mediate the pathological response. This study follows on from the GWAS findings that a non synonymous SNP in the coding region of *TNS1* is associated with COPD risk. We therefore assessed the roles and interactions of tensin1 in healthy, asthmatic and COPD airways. We have demonstrated for the first time that tensin1 is expressed in the apical airway epithelium, ASM bundles and airway lamina propria in both non-COPD control and COPD lung resections, and healthy and asthmatic endobronchial biopsies. A significant increase in tensin1 immunostaining was detected in the ASM and lamina propria in COPD subjects when compared to non-COPD controls, but no differences were evident between asthma and health. Tensin1 mRNA and protein expression were present in cultured HASMCs. Of note, tensin1 co-localised and interacted physically with αSMA in actin stress fibres, and influenced αSMA expression, as tensin1 silencing led to downregulation of αSMA protein. As a result, tensin1 appears to make a critical contribution to the contractile properties of HASMCs.

Tensin1 protein expression was upregulated in the ASM bundles in COPD compared to non-COPD controls, but not in cultured HASMCs, suggesting that the *in vivo* findings are regulated by local factors that are lost in cell culture. TGFβ1 is a candidate molecule regulating this *in vivo* as it is upregulated in COPD airways^24^, and we found that tensin1 mRNA expression was markedly upregulated by TGFβ1 in HASMCs *in vitro*. This is in keeping with the effects of TGFβ1 on tensin1 expression in parenchymal lung fibroblasts obtained from patients with idiopathic pulmonary fibrosis^25^. However, TGFβ1 did not have a detectable effect on total tensin1 expression in HASMCs, but instead altered its intracellular distribution promoting the formation of long tensin1-containing fibrillar adhesions. To account for the increased overall levels of tensin1 protein evident in COPD airways relative to asthma and healthy tissue biopsies, factors in addition to TGFβ1 are likely to contribute. These factors would be less critical in asthmatic than COPD patients. Candidate factors would include other pro-inflammatory mediators present in COPD, the airway response to cigarette smoke and associated epigenetic changes, or the response to impaired airway stretch present in COPD. These various hypotheses will need testing in future work, but may explain why tensin1 expression was not increased in asthmatic airways.

Tensins are known to play a key role in remodeling the extracellular matrix through their interactions with integrins^18,25^, and so the high expression in patients with COPD may be particularly relevant to the fixed airflow obstruction and small airway fibrosis present in COPD. This would be in keeping with the pro-fibrotic role of tensin1 identified in patients with idiopathic pulmonary fibrosis^25^. In addition, an actin-binding domain is located on the N-terminal of tensin1 enabling interaction with actin filaments of the cytoskeleton, specifically within fibrillar adhesions that cause cellular stiffening in IPF^25^. The co-localisation of tensin1 with αSMA within stress fibres, and the marked downregulation of αSMA following tensin1 silencing, suggest feedback between tensin1 gene transcription and/or protein expression and factors regulating αSMA gene regulation. The fact that there were differences in the demographics of the groups providing HASMCs for culture, but no difference in the way their HASMCs behave in culture, indicates that the role of tensin1 in ASM contraction is a fundamental role irrespective of disease status and patient characteristics. Our data do not permit relative quantitative comparisons of the direct contribution tensin1 makes to the contractile properties of HASMCs versus indirect effects through regulation of αSMA expression. However they indicate tensin1 is associated with αSMA transcription and, potentially, retention of the protein product within the cell.

Having investigated the expression and function of tensin1 in COPD, we next examined the association and functional effects of the COPD-associated SNP in the *TNS1* gene, identified in GWAS^14,13^. The polymorphism is a non synonymous SNP (rs2571445) in the *TNS1* gene and the base pair change encoded by this SNP leads to the amino acid substitution R1197W. The SNP is present in approximately 40% of the population, and its presence is associated with reduced lung function in GWAS^14^. It was therefore remarkable, that in spite of the relatively small number of subjects studied here, there was very strong segregation between homozygotes for C/C (which generates R1197) who were largely healthy, and patients with COPD or asthma who were predominantly heterozygous C/T or occasionally homozygous T/T (R1197W). These data lend support to the GWAS findings. They suggest that R1197 in tensin1 is potentially protective against the development of both COPD and asthma, and/or that R1197W is a factor promoting the development of COPD and asthma. The exact role that R1197W plays in COPD susceptibility remains unclear as we were unable to find any differences in gene expression or survival between cells expressing R1197W and those homozygous for R1197.

Tensin1 protein expression was increased in the ASM bundles in COPD patients, but not in asthma, indicating that its increased expression is associated with marked fixed airflow obstruction. These data suggest that tissue factors regulating tensin1 turnover may be important. The R1197W mutation is predicted to alter the protease sensitivity of the protein, and *ex vivo* samples supported differential tensin1 cleavage profiles in HASMCs derived from healthy and COPD tissue. We therefore hypothesize that the R1197W mutation and COPD-specific co-factors together modulate tensin1 degradation and thereby affect αSMA expression, and ASM mass (Fig. 6F, schema). This would rationalise the different observations in tissue from asthmatic patients.

In conclusion, we have demonstrated immunoreactivity for tensin1 in the ASM, lamina propria and airway epithelium in human lung tissue. Tensin1 expression was increased in the ASM and lamina propria in COPD donors when compared to non-disease controls, but was not increased in asthma. Tensin1 was enriched in fibrillar adhesions in HASMCs upon stimulation with TGFβ1 and fibronectin. This is likely of great importance as it allows tensin1 to interact with other proteins, leading to activation of signaling pathways and remodeling of the extracellular matrix. Furthermore tensin1 plays a critical role in regulating HASMC αSMA expression and contraction suggesting it may play an important role in both COPD and asthma pathophysiology.

## Methods

### Subjects

#### COPD and control tissue for immunohistochemistry

To study COPD airway tissue by immunohistochemistry, we used 2^nd^-5^th^ generation airway tissue collected at the time of lung resection for lung cancer. The tissue used was well demarcated from the tumour and not affected by the tumour process. The COPD group were all ex or current smokers with spirometric evidence of airflow obstruction and met Global Obstructive Lung Disease (GOLD) criteria for COPD^26^. Non-COPD control tissue was used from patients who did not have evidence of COPD (see Table 1).

#### Asthma and control tissue for immunohistochemistry

People with asthma and healthy controls underwent fibreoptic bronchoscopy as described previously. Subjects with a history of asthma had a < 10 pack year smoking history and met a diagnosis for asthma as described previously^20^ (see Table 2).

#### Asthma and COPD tissue for primary human airway smooth muscle culture

For the study of primary human ASM cells (HASMCs) in culture, healthy control subjects and subjects with COPD or asthma underwent fibreoptic bronchoscopy as described previously^20^(see Table 3), and HASMCs cultured as described below.

All research participants gave written informed consent, and the collection of tissue was approved by the National Research Ethics Service (reference numbers: 07/MRE08/42, 04/Q2502/74, 08/H0406/189). All methods were performed in accordance with the relevant guidelines and regulations.

### Human airway smooth muscle cell (HASMC) isolation and culture

Subjects underwent bronchoscopy, and mucosal biopsies were collected for HASMC culture as described previously^27^. Pure human ASM bundles in bronchial biopsy tissue (n = 30) (and one lung resection sample) were dissected free of surrounding tissue. The ASM bundles were cultured in DMEM supplemented with 10% FCS, 4 mM L-glutamine, 100 U/ml penicillin, 100 mg/ml streptomycin, and 0.25 μg/ml amphotericin. HASMC characteristics were determined by immunofluorescence with antibodies to α-smooth muscle actin (αSMA) (FITC directly conjugated) and myosin indirectly labelled with FITC^27^.

### Immunohistochemistry

Bronchial tissue remote from the cancer was dissected from the lung resection material and embedded in glycol‐methacrylate (GMA) and stored at −20 °C as described previously^28^. Bronchial biopsies taken at bronchoscopy were also embedded in GMA as described previously^28^. Two GMA sections of 2 μm thickness and at least 10 µm apart were immunostained for Tensin1 (SAB4200283, 2.5 μg/ml, Sigma-Aldrich) or the appropriate isotype control at the same concentration as the primary antibody (X0936, 2.5 µg/ml, Dako) using the Dako EnVsion FLEX + staining technique. Sections were counter-stained using Mayer’s haematoxylin and visualised using a light microscope. The tensin1 antibodies were validated further using immunoprecipitation and siRNA downregulation (see below).

Images of complete tissue sections were collected and tensin1 immunostaining was quantified by a blinded observer in the airway epithelium, lamina propria and airway smooth bundles using Cell^F^ software version 5.0 (Olympus). The thresholding technique was used to quantify tensin1 immunostaining based on the hue saturation and intensity (HSI) value as described previously^20^. Tensin1 immunostaining levels were correlated with smooth muscle bundle area measurements.

### Quantitative real time PCR

HASMC RNAs were isolated using the RNeasy Plus Kit (Qiagen, Manchester, UK) according to manufacturer’s instructions. Detection of *TNS1* and α*SMA* mRNA was performed using the Fast SYBR Green Master Mix, alongside primers targeting the internal normalised gene *β-actin*. PCR products were run on an agarose gel to confirm the product amplified was the correct size, and were also sequenced. *TNS1* and α*SMA* mRNA expression was quantified using the ΔC_T_ method^29^. Full experimental details can be found in the Supplementary Information.

Cells were stimulated with 10 ng/ml transforming growth factor-β1 (TGFβ1) (R&D systems), cells were grown to confluence and then serum-starved for 24 hours prior to stimulation for a further 24 hours.

### Western blotting

Tensin1 expression in primary HASMCs was analysed by western blotting as described previously^30^. HASMCs were disrupted in lysis buffer and soluble protein from equivalent number of cells was resolved by 7.5% SDS-page and then transferred to a polyvinylidene (PVDF) membrane. The membrane was blocked using 5% Milk +0.1% TBS Tween20 and incubated with antibodies to Tensin1 (SAB4200283, 1 μg/ml, Sigma-Aldrich), β-actin (sc-47778, 0.04 μg/ml Santa-Cruz) and αSMA (M0851, 1 µg/ml, Dako). Secondary antibodies conjugated to horseradish peroxidase (HRP) were then applied (goat anti-rabbit HRP [sc-2054, 0.08 μg/ml, Santa-Cruz] or goat anti-mouse HRP [P0447, 0.5 μg/ml, Dako]). Immunolabelled proteins were visualised by chemiluminescence using ECL substrate and the ImageQuant LA S 4000 (GE Healthcare Life Sciences, Little Chalfont, UK). Band intensity was quantified using ImageJ software (National Institutes of Health; http://rsbweb.nih.gov/ij/).

### Immunoprecipitation

HASMCs were disrupted in lysis buffer and as a pre-clearing step, incubated with Protein A/G beads (sc-2003, Santa-Cruz) for 30 minutes at 4 °C. Separately, Protein A/G beads were incubated with either anti-tensin1 antibody (SAB4200283, 4 µg/ml, Sigma-Aldrich or sc-28542, 2 µg/ml, Santa-Cruz) or isotype control rabbit IgG (X0936, 4 µg/ml, Dako) for 30 minutes at 4 °C. Pre-cleared lysates were then incubated with bead-antibody complexes for 16 hours at 4 °C. Immunoprecipitated complexes were washed three times in lysis buffer and once in PBS, eluted in Laemmli buffer, and denatured for 5 minutes at 95 °C. Western blot analysis was then performed. Immunoprecipitates were probed with two anti-tensin1 antibodies (Sigma-Aldrich [SAB4200283, 1 µg/ml] and Santa-Cruz [sc-28542, 4 µg/ml]) to examine tensin1 antibody specificity.

To examine tensin1-αSMA interaction, Dynabeads^TM^ Protein G (10003D, ThermoFisher) were used. Again, HASMCs were disrupted in lysis buffer and as a pre-clearing step, incubated with Protein G Dynabeads for 1 hour at 4 °C. Pre-cleared lysates were then incubated with either anti-tensin1 antibody (SAB4200283, 4 µg/ml, Sigma-Aldrich or sc-28542) or isotype control rabbit IgG (X0936, 4 µg/ml, Dako) for 4 hours at 4 °C. Lysate-antibody complexes were then incubated with Protein G Dynabeads for 16 hours at 4 °C. Immunoprecipitated complexes were washed three times in lysis buffer and once in PBS, eluted in Laemmli buffer at room temperature for 30 minutes. Western blot analysis was then performed. Immunoprecipitates were probed with anti-αSMA (M0851, 1 µg/ml, Dako).

### Immunofluorescence

HASMCs were seeded into 8-well chamber slides, grown to confluence, and immunostained using mouse monoclonal αSMA (0.7 µg/ml, Dako) and isotype control FITC-conjugated mouse IgG2a (X0933, 10 µg/ml, Dako, Ely, UK), anti-Tensin1 antibody (4.5 µg/ml, Sigma-Aldrich) and isotype control rabbit IgG (4.5 µg/ml, Dako). Secondary antibodies labelled with AlexaFluor594 (A-11012, ThermoFisher) or FITC (FO382, Sigma-Aldrich) were applied and the cells were counterstained with 4′, 6-diamidino-2-phenylindole (DAPI) (32670, Sigma-Aldrich). To study the effect of the extracellular matrix protein fibronectin and TGFβ1 on tensin1 expression, slides were coated with human recombinant fibronectin (F0895, Sigma-Aldrich) for 1 hour prior seeding and stimulated with TGFβ1 (10 ng/ml) for 24 hours. When 50% confluent, cells were stained as above and mounted with fluorescent mounting medium. Original images were captured on a confocal immunofluorescence microscope (Leica TCS SP5, UK) and staining was quantified using Cell F imaging software (Olympus UK Ltd). Matched exposures were used for isotype controls. Quantification of the co-localisation of tensin1 and αSMA proteins was performed using an Image J plugin, JaCoP, in which Mander’s overlap coefficient and Pearson’s correlation were calculated (National Institutes of Health; http://rsbweb.nih.gov/ij/). Quantification of fibrillar adhesion length was measured by Cell F imaging software (Olympus UK Ltd.).

### Human airway smooth muscle cell transfection with siRNA

HASMCs were transfected with siRNA smartpools directed against *TNS1* (M-009976-00, Dharmacon) and a non-targeting siRNA control (D-001206-14, Dharmacon). HASMCs were plated in culture media in the absence of antibiotics in a 6 well plate and incubated overnight. Lipofectamine 2000 (11668, Invitrogen) and siRNA mixture was added to cells. The cells were incubated with the complexes for 5 hours. After 5 hours, medium was replaced with antibiotic-free media for 48 hours^31^. Tensin1 siRNA-transfected HASMCs derived from healthy and COPD individuals along with controls cells (untreated cells, transfection reagent alone, non-targeting siRNA control) were immunostained for both tensin1 (4.5 µg/ml, Sigma-Aldrich) and αSMA (0.7 µg/ml, Dako). Cells were also stimulated with TGFβ1 (10 ng/ml) to examine its role on αSMA expression after silencing tensin1. Full experimental details can be found in the Supplementary information.

### Survival/proliferation assay

The MTS assay (G3582, Promega) was used to assess survival and proliferation of the cells after tensin1 silencing. 48 hours after transfection, cells were collected and plated into 96-well plates in culture media in the absence of antibiotics overnight. Cells were stimulated with TGFβ1 for 24 hours. 20 μl of MTS solution was added to each well. Plates were then incubated at 37 °C for 4 hours. The optical density (OD) at 490 nm was determined with a spectrophotometer. Each experimental condition was run in triplicate.

### TGFβ1 ELISA

Tensin1 knockdown was performed as described above and supernatants were collected. A commercial ELISA was used to measure TGFβ1 in tensin1 knockdown sample supernatants according to the manufacturer’s protocol (DY240, R&D systems). Full experimental details can be found in the Supplementary Information.

### Collagen gel contraction assay

Tensin1 knockdown was performed as above and then cells were detached and embedded in collagen gels as described previously^32^. Bradykinin was then added to appropriate wells to a final concentration of 1 nM (B3259, Sigma-Aldrich). Photographs were taken at 0, 4, 18, 24 and 48 hours. The surface area was measured at each time point using ImageJ software (National Institutes of Health; http://rsbweb.nih.gov/ij/).

### SNP genotyping

PCR-restriction fragment length polymorphism (RFLP) analysis was used to genotype cultured cells for the presence of the polymorphism rs2571445. Full experimental details are provided in the Supplementary Information (can be found on the Supplementary information). The homozygote T/T genotype produced one band (278 bp), the homozygote C/C genotype produced two bands (214 and 64 bp) and the heterozygote C/T genotype displayed all three bands (278, 214 and 64 bp).

### Statistical analysis

Data distribution was tested for normality using the Kolmogorov-Smirnov test. Data across groups were compared with either the ANOVA or Kruskall Wallis tests where appropriate. Between group comparisons were analysed using Dunnett’s/Tukey’s multiple comparison test, or Students unpaired/paired *t* test, or Mann Whitney U/Wilcoxon Signed Rank for paired and unpaired parametric and non-parametric data, respectively. Data were analysed with GraphPad Prism 6 (GraphPad Software, Inc., La Jolla, CA, USA). P < 0.05 was taken as statistically significant.

### Ethical approval and consent to participate

All subjects gave written informed consent, and the collection of tissue was approved by the National Research Ethics Service. Reference numbers: 07/MRE08/42, 04/Q2502/74 and 08/H0406/189.

## Supplementary information

Supplementary information

## Data Availability

All data generated or analysed during this study are included in this published article [and its Supplementary Information Files].
