# Supplementary material for: Tensin1 expression and function in chronic obstructive pulmonary disease
Source: Sci Rep. 2019 Dec 12;9:18942. doi: 10.1038/s41598-019-55405-2 (PMC6908681; doi:10.1038/s41598-019-55405-2)
Supplement: Supplementary file 1 — Supplementary information [file 41598_2019_55405_MOESM1_ESM.pdf]

Tensin1 expression and function in chronic obstructive pulmonary disease

Panayiota Stylianou, PhD[1], Katherine Clark, PhD[2], Bibek Gooptu, PhD[1,3], Dawn Smallwood, PhD[4], Christopher E. Brightling, PhD[1], Yassine Amrani, PhD[1], Katy M. Roach, PhD[1], Peter Bradding, DM[1*]

[1]Dept of Respiratory Sciences, University of Leicester, UK, Institute of Lung Health and NIHR Leicester BRC-Respiratory, UK

[2]Dept of Molecular and Cell Biology, University of Leicester, UK

[3]Leicester Institute of Structural and Chemical Biology, University of Leicester, UK

[4]Faculty of Health and Life Sciences, De Montfort University, UK

Corresponding author

Professor Peter Bradding, Department of Respiratory Sciences, Glenfield Hospital, Groby Rd, Leicester, LE3 9QP UK. Tel +44 116 2583998 Fax +44 116 2502787, E-mail: pb46@le.ac.uk

Contributing authors

Dr Panayiota Stylianou, Department of Respiratory Sciences, Glenfield Hospital, Groby Rd, Leicester, LE3 9QP UK. Tel +44 116 2583024, E-mail: ps401@le.ac.uk

Dr Katherine Clark, Department of Molecular and Cell Biology, Henry Welcome Building, Lancaster road, Leicester, LE1 7HB UK. Tel +44 116 252 3455, E-mail: kc57@le.ac.uk

Professor Bibek Gooptu, Department of Respiratory Sciences, Glenfield Hospital, Groby Rd, Leicester, LE3 9QP UK & Leicester Institute of Structural and Chemical Biology, Henry Welcome Building, Lancaster road, Leicester, LE1 7HB UK. Tel +44 116 229 7137, E-mail: bg129@le.ac.uk

Dr Dawn Smallwood, Leicester School of Allied Health Sciences, Faculty of Healthy and Life Sciences, Hawthorn Building, De Montfort University, Leicester, LE1 9BH UK. Tel +44 116 257 7469, E-mail: dawn.smallwood@dmu.ac.uk

Professor Christopher E Brightling, Department of Respiratory Sciences, Glenfield Hospital, Groby Rd, Leicester, LE3 9QP UK. Tel +44 116 258 3998, E-mail: ceb17@le.ac.uk

Dr Yassine Amrani, Department of Respiratory Sciences, Glenfield Hospital, Groby Rd, Leicester, LE3 9QP UK. Tel +44 116 258 3694, E-mail: ya26@le.ac.uk

Dr Katy M Roach, Department of Respiratory Sciences, Glenfield Hospital, Groby Rd, Leicester, LE3 9QP UK. Tel +44 116 258 3034, E-mail: kmr11@le.ac.uk

METHODS

Subjects

For the study of human airway smooth muscle cells (HASMCs) in culture, healthy control subjects and subjects with COPD or asthma were recruited from respiratory clinics and underwent bronchoscopy. COPD subjects were all ex or current smokers with spirometric evidence of airflow obstruction according to the Global Obstructive Lung Disease (GOLD) criteria for COPD [1]. Subjects with a history of asthma had a <10 pack year smoking history and met a diagnosis for asthma as described previously [2]. Control subjects included those with and without a smoking history without airflow obstruction.

To study tissue by immunohistochemistry, we used two sources of tissue; i) airway tissue collected at the time of lung resection from lung cancer patients who also met the GOLD criteria for COPD. Control tissue was also collected from subjects undergoing lung resection without evidence of COPD; ii) bronchial biopsies from healthy volunteers and asthmatic patients as described for HASMC cell culture [2, 3]. All subjects gave written informed consent, and the collection of tissue was approved by the National Research Ethics Service (reference numbers: 07/MRE08/42, 04/Q2502/74, 08/H0406/189). All methods were performed in accordance with the relevant guidelines and regulations.

Human airway smooth muscle cell (HASMC) isolation and culture

Subjects underwent bronchoscopy, and mucosal biopsies were collected for HASMC culture as described previously [3]. Pure human airway smooth muscle (ASM) bundles in airways isolated from lung resection and bronchial biopsy tissue were dissected free of surrounding tissue. The ASM bundles were cultured in DMEM supplemented with 10% FCS, 4 mM L-glutamine, 100 U/ml penicillin, 100mg/ml streptomycin, and 0.25μg/ml amphotericin. HASMC characteristics were determined by immunofluorescence with antibodies to α-smooth muscle actin (αSMA) (FITC directly conjugated) and myosin indirectly labelled with FITC [3].

Immunohistochemistry

Bronchial tissue remote from the cancer was dissected from the lung resection material and embedded in glycol-methacrylate (GMA) and stored at −20°C as described previously [4]. Bronchial biopsies taken at bronchoscopy were also embedded in GMA as described previously [4]. GMA sections of 2 µm thickness were immunostained for Tensin1 (SAB4200283, 2.5 µg/ml, Sigma-Aldrich and sc-28542, 1 µg/ml, Santa-Cruz) or the appropriate isotype control at the same concentration as the primary antibody (X0936, 2.5 µg/ml, Dako) using the Dako EnVsion FLEX+ staining technique. Sections were counter-stained using Mayer's haematoxylin and visualized using a light microscope. The tensin1 antibodies were validated further using immunoprecipitation and siRNA downregulation (see below).

Images were collected and tensin1 immunostaining was quantified using Cell^{F} software version 5.0 (Olympus). The thresholding technique was used to quantify tensin1 immunostaining based on the hue saturation and intensity (HSI) value [2].

Quantitative Real Time PCR

HASMC and HBEC RNAs were isolated using the RNeasy Plus Kit (Qiagen, Manchester, UK) according to manufacturer's instructions. Detection of TNS1 mRNA was performed using the Fast SYBR Green Master Mix (Applied Biosystems, Paisley, UK) (TNS1 (forward primer: 5'-AGCGGAGACCTGACATCAC-3', reverse primers: 5'-CGGTTTCCCTTGTTGTGTAGAAC-3') and αSMA (forward primer: 5'-GAAGGAATAGCCACGCTCAG-3', reverse primer 5'-TTCAATGTCCCAGCCATGTA-3')), alongside primers targeting the internal normalized gene β-actin (Hs_ACTB_1_SG Quantitect Primer Assay, QT00095431; Qiagen). PCR products were run on an agarose gel to confirm the product amplified was the correct size, and were also sequenced. Tensin1 and αSMA mRNA expression was quantified using the ΔC_T method [5].

Cells were stimulated with 10 ng/ml transforming growth factor-β1 (TGFβ1) (R&D systems), cells were grown to confluence and then serum-starved for 24 hours prior to stimulation for a further 24 hours.

Western blotting

Tensin1 expression in primary HASMCs was analysed by western blotting as described previously [6]. HASMCs were disrupted in lysis buffer and soluble protein from equivalent number of cells was resolved by 7.5% SDS-page and then transferred to a polyvinylidene (PVDF) membrane. The membrane was blocked using 5% Milk +0.1% TBS Tween20 and incubated with antibodies to Tensin1 (SAB4200283, 1 μg/ml, Sigma-Aldrich) and β-actin (sc-47778, 0.04 μg/ml Santa-Cruz) and αSMA (M0851, 1μg/ml, Dako). Secondary antibodies conjugated to horseradish peroxidase (HRP) were then applied (goat anti-rabbit HRP [sc-2054, 0.08 μg/ml, Santa-Cruz] or goat anti-mouse HRP [P0447, 0.5 μg/ml, Dako]). Immunolabelled proteins were visualized by chemiluminescence using ECL substrate and the ImageQuant LA S 4000 (GE Healthcare Life Sciences, Little Chalfont, UK). Band intensity was quantified using ImageJ software (National Institutes of Health; http://rsbweb.nih.gov/ij/).

Immunoprecipitation

HASMCs were disrupted in lysis buffer and as a pre-clearing step incubated with Protein A/G beads (sc-2003, Santa-Cruz) for 30 minutes at 4°C. Separately Protein A/G beads were incubated with either anti-tensin1 antibody (SAB4200283, 4 μg/ml, Sigma-Aldrich or sc-28542, 2 μg/ml, Santa-Cruz) or isotype control rabbit IgG (X0936, 4 μg/ml, Dako) for 30 minutes at 4°C. Pre-cleared lysates were then incubated with bead-antibody complexes for 16 hours at 4°C. Immunoprecipitated complexes were washed three times in lysis buffer and once in PBS, eluted in Laemmli buffer and denatured for 5 minutes at 95°C. Western blot analysis was then performed. Immunoprecipitates were probed with two anti-tensin1 antibodies (Sigma-Aldrich (SAB4200283, 1 μg/ml) and Santa-Cruz (sc-28542, 4 μg/ml)) and anti-αSMA (M0851, 1μg/ml, Dako) to examine tensin1 antibody specificity and the tensin1-αSMA interaction respectively.

Immunofluorescence

HASMCs were seeded into 8-well chamber slides, grown to confluence, and immunostained using mouse monoclonal αSMA (0.7 μg/ml, Dako) and isotype control FITC-conjugated mouse IgG2a

(X0933, 10 μg/ml, Dako, Ely, UK), anti-Tensin1 antibody (4.5μg/ml, Sigma-Aldrich) and isotype control rabbit IgG (4.5μg/ml, Dako). Secondary antibodies labelled with AlexaFluor594 (A-11012, ThermoFisher) or FITC (FO382, Sigma-Aldrich) were applied and the cells were counterstained with 4', 6-diamidino-2-phenylindole (DAPI) (32670, Sigma-Aldrich). To study the effect of the extracellular matrix protein fibronectin and TGFβ1 on tensin1 expression, slides were coated with human recombinant fibronectin (F0895, Sigma-Aldrich) for 1 hour prior seeding and stimulated with TGFβ1 (10ng/ml) for 24 hours. When 50% confluent, cells were stained as above and mounted with fluorescent mounting medium. Original images were captured on a confocal immunofluorescence microscope (Leica TCS SP5, UK) and staining was quantified using Cell F imaging software (Olympus UK Ltd). Matched exposures were used for isotype controls. Quantification of the co-localisation of tensin1 and αSMA proteins was performed using an Image J plugin, JaCoP, in which Mander's overlap coefficient and Pearson's correlation were calculated (National Institutes of Health; http://rsbweb.nih.gov/ij/). Quantification of fibrillar adhesion length was measured by Cell F imaging software (Olympus UK Ltd).

Human airway smooth muscle cell transfection with siRNA

HASMCs were transfected with siRNA smartpools directed against tensin1 (M-009976-00, Dharmacon) and a non-targeting siCONTROL (D-001206-14, Dharmacon). HASMCs were plated in culture media in the absence of antibiotics in a 6 well plate and incubated at 37°C, 5% CO_2 overnight. 5 μl of Lipofectamine 2000 (11668, Invitrogen) was diluted in 105 μl serum and antibiotic-free media and incubated for 5 minutes before adding to siRNA diluted in 105 μl of serum and antibiotic-free media. The mixture was incubated for 20 minutes and added to cells with an additional 1.78 ml culture media. The cells were incubated with the complexes for 5 hours. After 5 hours, medium was replaced with antibiotic-free media for 48 hours [7].

Survival/Proliferation assay

The MTS assay (G3582, Promega) was used to assess survival and proliferation of the cells after tensin1 silencing. 48 hours after transfection, cells were collected and 2 x 10^3 cells were plated into 96-well plates in culture media in the absence of antibiotics overnight. Cells were stimulated with TGFβ1 for 24 hours. 20 μl of MTS solution was added to each well. Plates were then incubated at 37°C for 4 hours. The optical density (OD) at 490nm was determined with a spectrophotometer. Each experimental condition was run in triplicate.

TGFβ1 ELISA

Tensin1 knockdown was performed as described above and supernatants were collected. A commercial ELISA was used to measure TGFβ1 in tensin1 knockdown sample supernatants according to the manufacturer's protocol (DY240, R&D systems). The lower limit of TGFβ1 detection was 0.031 pg/ml and the upper limit was 2 pg/ml. Assay samples were diluted and activated before ELISA. Each experimental condition was run in triplicate.

Collagen gel contraction assay

Tensin1 knockdown was performed as above and then cells were detached and embedded in collagen gels as described previously [8]. Bradykinin was then added to appropriate wells to a final concentration of 1nM (B3259, Sigma-Aldrich). Photographs were taken at 0, 4, 18, 24 and 48 hours. The surface area was measured at each time point using ImageJ software (National Institutes of Health; http://rsbweb.nih.gov/ij/).

pEGFP *TNS1* constructs

pEGFP construct containing full length *TNS1* with the C allele was provided by Dr Katherine Clark and used for validation of the RFLP genotyping method [9]. pEGFP construct containing full length *TNS1* with the T allele was generated. Site-directed mutagenesis was performed using the following primer sequences: forward 5'-CTAGTCCTGGCTTCGGCTGGCGG-3' and reverse 5'-CGGCTGGCGGGCCATCAATCCC-3'. These primers were used to introduce mutation and amplify cDNA

by PCR, using Platinum Pfx DNA polymerase (Invitrogen), from the pEGFP construct containing full length *TNS1* with the C allele. The amplified material was digested with appropriate restriction enzymes and cloned into pEGFP–C1 clone (ClonTech). Final product was sequenced to confirm identity and introduction of the mutation.

SNP genotyping

PCR-restriction fragment length polymorphism (RFLP) analysis was used to genotype cultured cells for the presence of the polymorphism rs2571445. The polymorphic region was amplified using the following primer sequences: forward 5'-ATCTGCCCAGAGAAACTACCAGAGC-3' and reverse 5'-ATCATCTGGTGATGGCTCAAACTG-3'. The enzyme Eag1 (ER0331, Invitrogen) was used to identify the different genotypes. First, PCR amplification was performed and then PCR products were digested with Eag1 for 1 hour at 37°C. The cleaved products were run on a 2.5% agarose gel and identified by ethidium bromide staining. A non-restricted digest PCR product was run for every subject. The homozygote T/T genotype produced one band (278bp), the homozygote C/C genotype produced two bands (214 and 64bp) and the heterozygote C/T genotype displayed all three bands (278, 214 and 64bp).

Statistical analysis

Data distribution was tested for normality using the Kolmogorov-Smirnov test. Data across groups were compared with either the ANOVA or Kruskall Wallis tests where appropriate. Between group comparisons were analysed using Dunnett's/Tukey's multiple comparison test, or Students unpaired/paired *t* test, or Mann Whitney U/Wilcoxon Signed Rank for paired and unpaired parametric and non-parametric data, respectively. Data were analyzed with GraphPad Prism 6 (GraphPad Software, Inc., La Jolla, CA, USA). $P < 0.05$ was taken as statistically significant.

REFERENCES

1 Strategy G. *From the Global Strategy for the Diagnosis, Management and Prevention of COPD, Global Initiative for Chronic Obstructive Lung Disease (GOLD) 2016.*, <http://goldcopd.org/> (2016).

2 Shikotra, A. *et al.* Increased expression of immunoreactive thymic stromal lymphopoietin in patients with severe asthma. *J Allergy Clin Immunol* **129**, 104-111.e101-109, doi:10.1016/j.jaci.2011.08.031 (2012).

3 Brightling, C. E. *et al.* The CXCL10/CXCR3 axis mediates human lung mast cell migration to asthmatic airway smooth muscle. *Am J Respir Crit Care Med* **171**, 1103-1108, doi:10.1164/rccm.200409-1220OC (2005).

4 Bradding, P. *et al.* Interleukin 4 is localized to and released by human mast cells. *J Exp Med* **176**, 1381-1386 (1992).

5 Livak, K. J. & Schmittgen, T. D. Analysis of relative gene expression data using real-time quantitative PCR and the 2(-Delta Delta C(T)) Method. *Methods* **25**, 402-408, doi:10.1006/meth.2001.1262 (2001).

6 Hollins, F. *et al.* Human airway smooth muscle promotes human lung mast cell survival, proliferation, and constitutive activation: cooperative roles for CADM1, stem cell factor, and IL-6. *J Immunol* **181**, 2772-2780 (2008).

7 Philips, P. A. (American Pancreatic Association, Pancreapedia: Exocrine Pancreas Knowledge Base, 2011).

8 Woodman, L. *et al.* Mast cells promote airway smooth muscle cell differentiation via autocrine up-regulation of TGF-beta 1. *J Immunol* **181**, 5001-5007 (2008).

9 Clark, K. *et al.* Tensin 2 modulates cell contractility in 3D collagen gels through the RhoGAP DLC1. *J Cell Biochem* **109**, 808-817, doi:10.1002/jcb.22460 (2010).

Figure S1

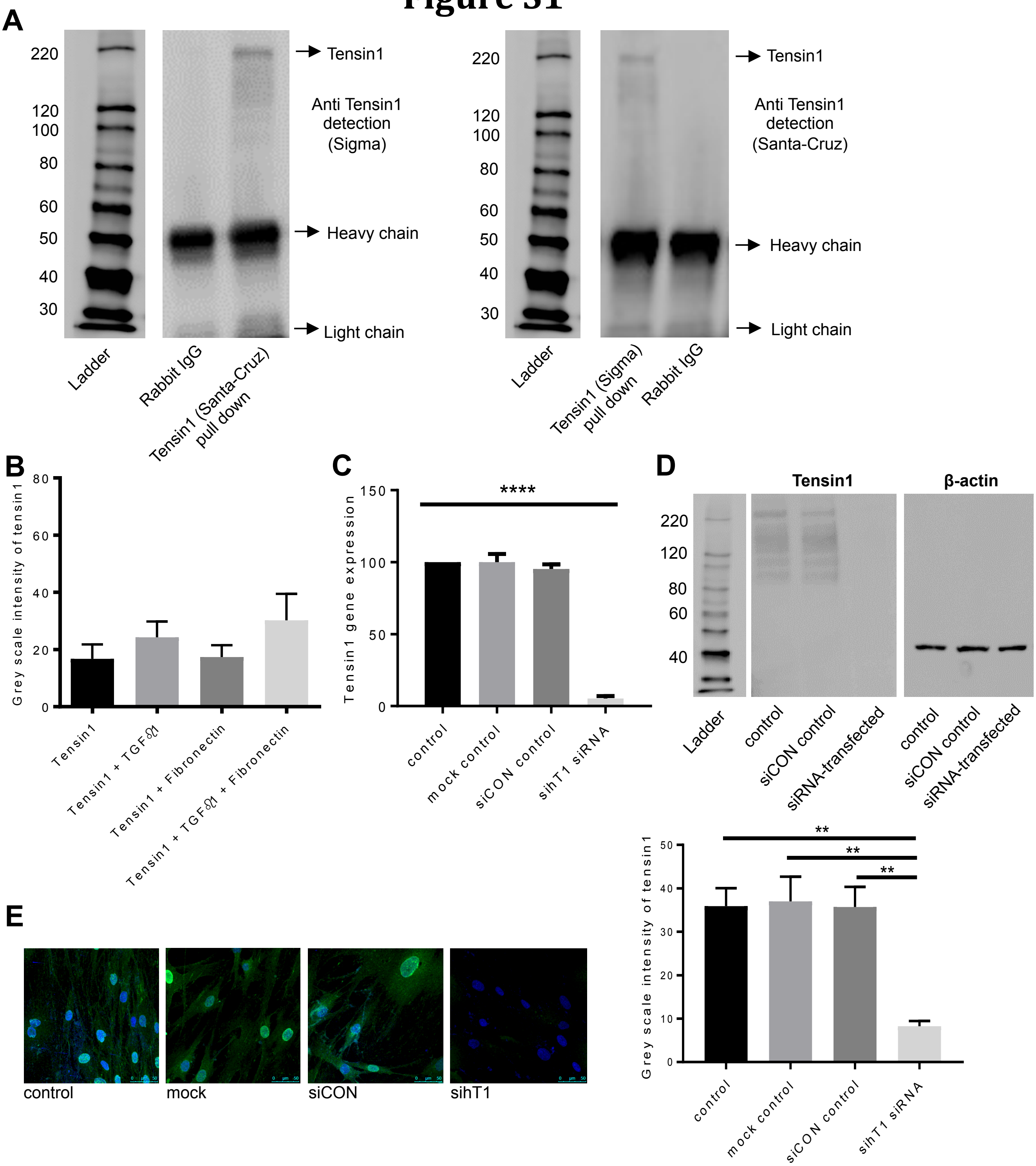

Figure S1. Tensin1 protein expression in HASMCs in health and COPD. (**A**) Immunoprecipitation using two tensin1 antibodies (from Sigma-Aldrich and Santa-Cruz). A single band of ~220kDa molecular weight was detected with both antibodies illustrating their specificity for tensin1. (**B**) Stimulation with TGFβ1 and fibronectin did not significantly increase the grey scale intensity of tensin1(mean±sem) (Dunnett's multiple comparison test as part of one-way ANOVA). (**C**) Silencing of tensin1 mRNA in HASMCs using siRNA. Approximately 92% downregulation was achieved (n=3) (mean±sem, ****p=0.0001) (Dunnett's multiple comparison test as part of one-way ANOVA). (**D**) A representative western blot from a COPD donor from the experiment in (C) is shown. (**E**) Silencing of tensin1 in HASMCs assessed using immunofluorescence analysis. Significant downregulation was achieved on 3 healthy and 3 COPD donors (Mean±SEM, **p=0.0030) (Dunnett's multiple comparison test as part of one-way ANOVA). Data in the right panel are pooled COPD and healthy controls which did not differ.

Figure S2

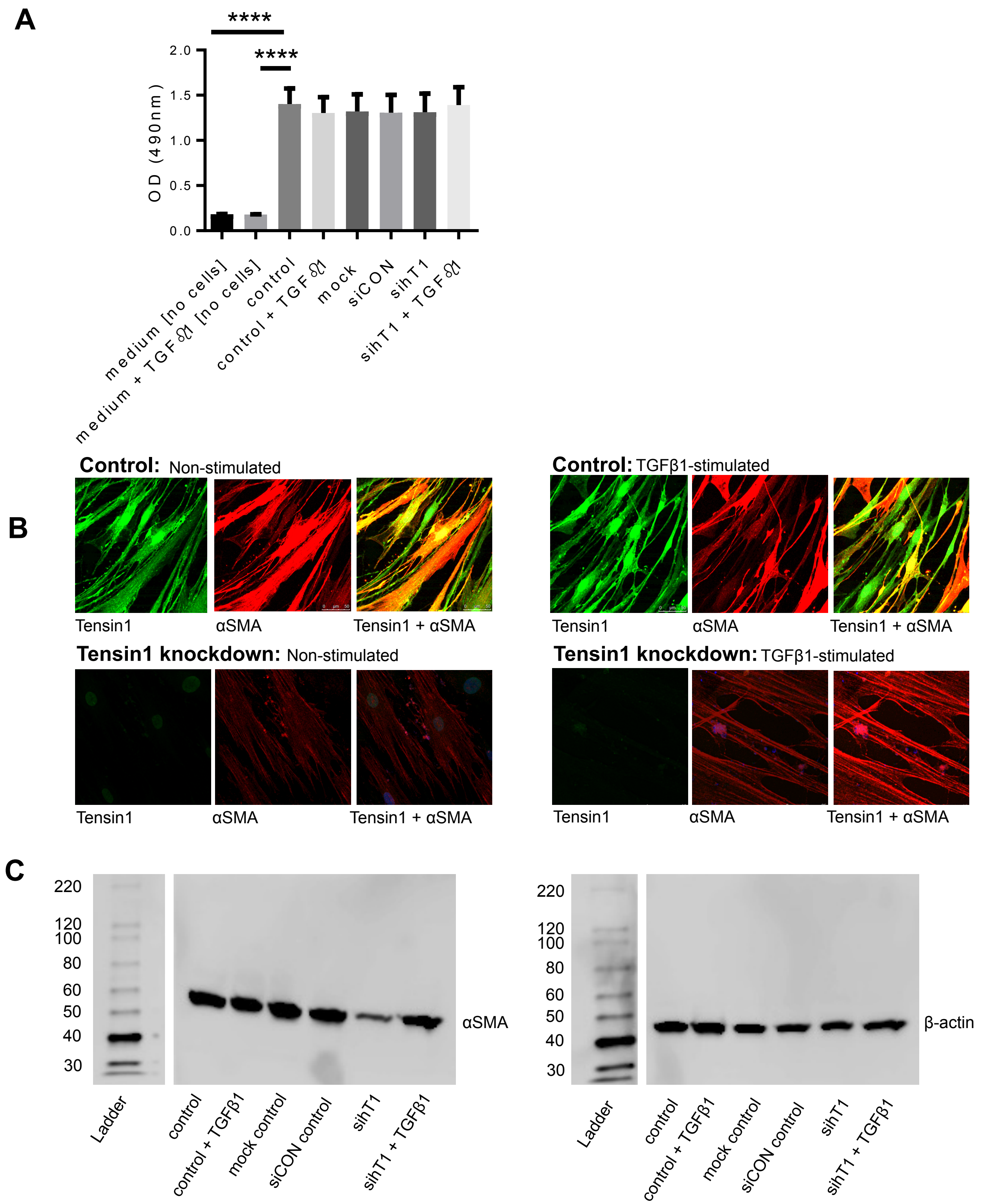

Figure S2. The effect of tensin1 silencing on αSMA expression in HASMCs derived from healthy and COPD subjects. (A) Cells were transfected with siRNA directed against tensin1. After tensin1 silencing was achieved, cell survival was assessed using the MTS assay. Data were compared using one-way ANOVA with Dunnett's multiple comparison test. Knockdown of tensin1 did not have any effect on HASMC survival when compared to the controls. Data shown are pooled from COPD (n=4) and healthy donors (n=4) which did not differ (Mean±SEM). **(B)** Cells were transfected with siRNA directed against tensin1 and assessed for αSMA expression using immunofluorescence analysis. A representative example showing decreased αSMA expression following tensin1 silencing when compared to controls (αSMA shown in red). **(C)** Full-length western blots of αSMA and β–actin showing in Figure 4c.

Figure S3

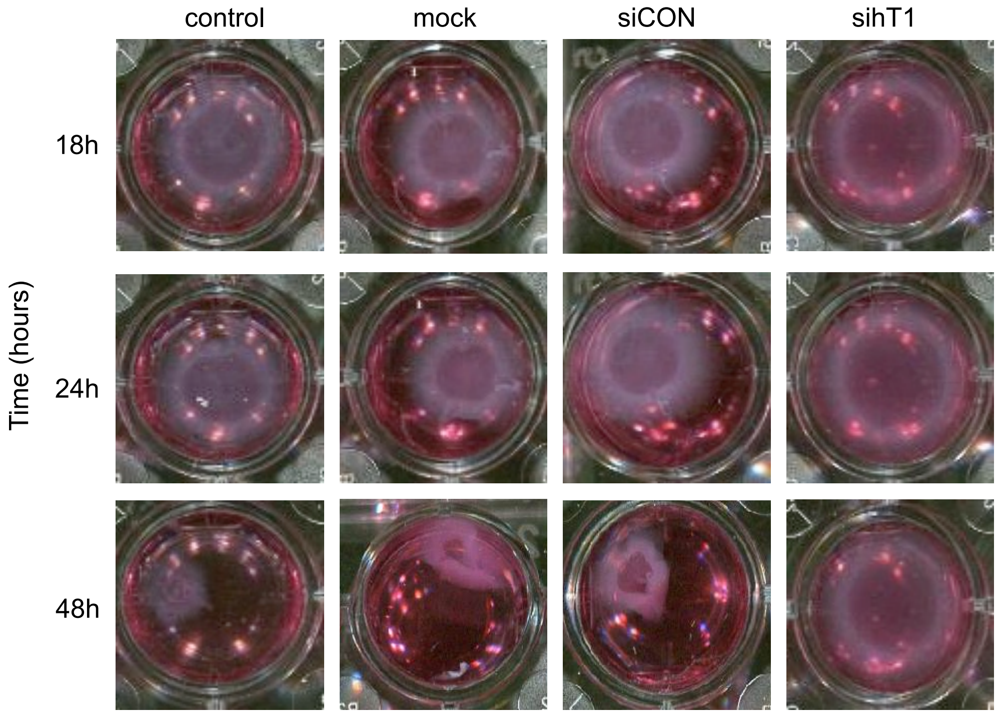

Figure S3. Collagen gel contraction by HASMC is dependent on tensin1. Cells were transfected with siRNA directed against tensin1 and incubated within 3D collagen gels. The extent of spontaneous collagen gel contraction was recorded at 4, 18, 24 and 48 hours. Assays were performed on 4 healthy and 4 COPD donors. Gels containing siRNA SMARTpool (sihT1) HASMCs showed a greatly reduced ability to contract, with significant differences compared to controls at 18 , 24 and 48 hours.

Figure S4

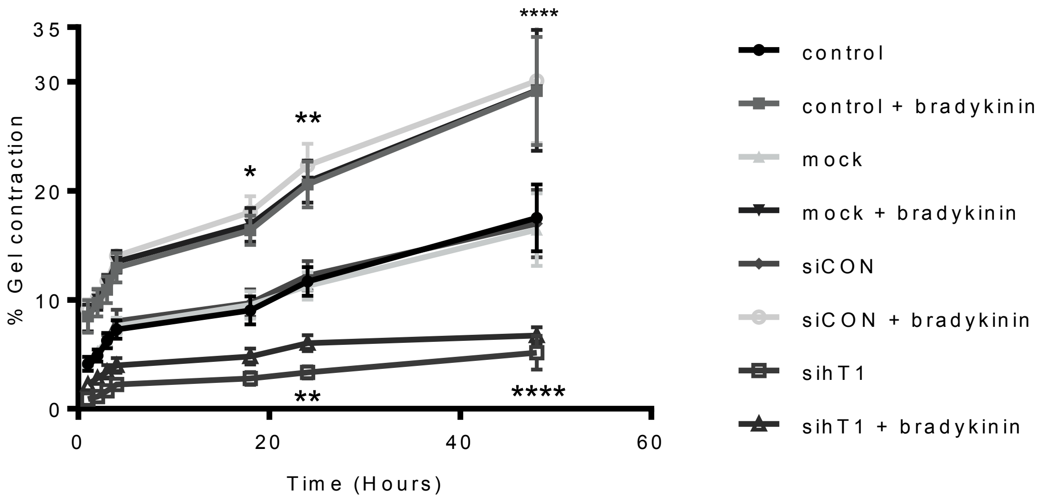

Figure S4. The effect of bradykinin on HASMC collagen gel contraction. Cells were transfected with siRNA directed against tensin1 and incubated within 3D collagen gels. The extent of spontaneous collagen gel contraction was recorded at 1,2,3, 4, 18, 24 and 48 hours. Quantification of collagen gel contraction using gel area measurement was performed (n=8, Mean±SEM). Data shown are pooled COPD (n=4) and healthy donors (n=4) which did not differ. HASMCs transfected with tensin1 siRNA SMARTpool showed a greatly reduced ability to contract, with significant differences compared to controls at 24 (**p=0.0052) and 48 hours (****p<0.0001). The extent of collagen gel contraction following bradykinin stimulation was significantly increased, with significant differences at 18 (non-transfected (*p=0.0187), mock (*p=0.0102) and siCON (**p=0.0020)) , 24 (non-transfected (**p=0.0023), mock (**p=0.0016) and siCON (***p=0.0001)) and 48 hours (non-transfected (****p<0.0001), mock (****p<0.0001) and siCON (****p<0.0001)). Tensin1-depleted HASMCs did not respond significantly to bradykinin, when compared to spontaneous contraction. Data analysed using two-way ANOVA with Dunnett's comparison test.

Figure S5

A

```
Query  827   AGGCGCGCCACAGAACAGTGGGCACCAACACTCCCCCTAGTCCTGGCTTCGGCTGGCGGG  886
             |||||||||||||||||||||||||||||||||||||||||||||||||||||| ||||||
Sbjct  4369  AGGCGCGCCACAGAACAGTGGGCACCAACACTCCCCCTAGTCCTGGCTTCGGCCGGCGGG  4428

Query  887   CCATCAATCCCAGCATGGCTGCCCCCAGCAGTCCCAGTTTGAGCCATCACCAGATGATGG  946
             ||||||||||||||||||||||||||||||||||||||||||||||||||||||||||||
Sbjct  4429  CCATCAATCCCAGCATGGCTGCCCCCAGCAGTCCCAGTTTGAGCCATCACCAGATGATGG  4488
```

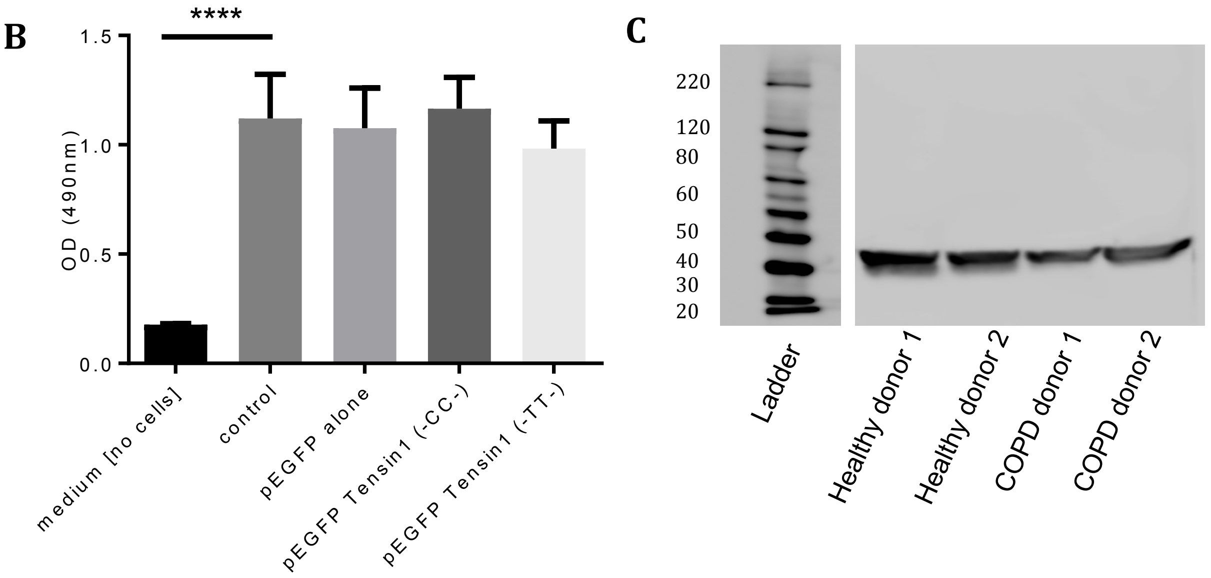

Figure S5. **Overexpressing tensin1 has no effect on proliferation**. (**A**) Sequencing data confirming insertion of mutation at 4422 bp and therefore generation of the pEGFP construct containing tensin1 (-CC-) used to validate the RFLP method and transfect HASMCs. (**B**) After tensin1 overexpression with pEGFP constructs containing full length of *TNS1* cDNA with either the C or T allele was achieved, cell survival and proliferation was assessed using the MTS assay. Data were compared using one-way ANOVA with Dunnett's multiple comparison test. Overexpression of tensin1 using pEGFP constructs did not have any effect on HASMC survival when compared to the controls. Data shown are pooled from COPD (n=4) and healthy donors (n=4) which did not differ (Mean±SEM). (**C**) Full-length western blot of β–actin shown in Figure 6e.
